# Sociotechnical Challenges in Implementing Domestic Violence Screening via Telehealth and In-Person Care: Qualitative Study on Clinicians’ Perspectives

**DOI:** 10.2196/75244

**Published:** 2025-10-23

**Authors:** Diana Freed, Marianne Sharko, Royoung Kim, Kelly Bartlett, Ermira Uldedaj, Natalie Bazarova, Lauri Goldkind

**Affiliations:** 1 Department of Computer Science Brown University Providence, RI United States; 2 Population Health Sciences Weill Cornell Medical College New York, NY United States; 3 New York Medical College Valhalla, NY United States; 4 New York City Mayor's Office to End Domestic and Gender-Based Violence New York, NY United States; 5 Cornell University Ithaca, NY United States; 6 Graduate School of Social Service Fordham University New York, NY United States

**Keywords:** domestic violence screening, health care stakeholders, clinical settings, telehealth, policy

## Abstract

**Background:**

New York State Public Health Law Section 2805-z (NYSPHL§2805-z) mandates that hospitals implement certain protocols for the identification of domestic violence (DV) to strengthen support for DV survivors. However, there are challenges within our current health care delivery settings that raise critical questions about the effectiveness and adaptability of mandated policies for DV screening. The COVID-19 pandemic significantly accelerated the adoption of telehealth services, leading to the widespread use of combined telehealth and in-person clinical care. This shift has brought challenges associated with implementing the mandated DV screening protocols.

**Objective:**

This study aimed to explore the challenges health care providers face in implementing DV screening that complies with NYSPHL§2805-z to support effective screening practices. Considering both the telehealth and in-person environment, we aimed to identify the challenges that health care providers face in meeting policy requirements, with a focus on the technical, environmental, and social factors impeding effective DV screening. We also explored potential sources of support to address these challenges. This study sought to provide actionable insights for enhancing policy implementation and improving care for DV survivors.

**Methods:**

We conducted interviews with health care professionals—nurses, social workers, and physicians—across New York City involved in DV screening. These interviews were analyzed using an adapted version of the Centers for Disease Control and Prevention sociotechnical model to understand how policy, organizational structures, and individual practices intersect and impact the effectiveness of DV screening, especially in telehealth settings.

**Results:**

Our findings revealed gaps in awareness of the policy and inconsistent DV screening processes. Through our interviews, we identified challenges in effective DV screening and strategies to improve the screening process. We found that the shift to telehealth introduced additional layers of complexity, with challenges in ensuring patient privacy and safety. Our findings revealed a lack of clear guidelines tailored to remote DV screenings and a need for training programs to prepare health care providers for the nuances of telehealth-based DV screening, both of which are crucial for complying with policy mandates. On the basis of this analysis, we developed a list of recommendations to support health care providers in effective screening for DV. Through user feedback, we confirmed that this list is consistent with the application of NYSPHL§2805-z among health care providers.

**Conclusions:**

As telehealth care is increasingly becoming an integral component of health care, there is an urgent need to refine and reinforce DV screening policies and procedures designed to comply with NYSPHL§2805-z. This study highlights the importance of developing practical and consistent telehealth protocols, enhancing provider training, creating supportive workflows, allotting adequate resources, and fostering collaboration among stakeholders to ensure that DV survivors receive the care they need in both remote and in-person clinical settings.

## Introduction

### Background

Domestic violence (DV) is defined by a persistent pattern of abusive and manipulative behavior within intimate relationships, aimed at gaining or maintaining power and coercive control over a partner [1]. This type of abuse manifests in various ways, including physical, sexual, emotional, financial, and technology-facilitated abuse. Health care professionals are crucial to identifying and supporting survivors of DV [2]. However, the success of these efforts is contingent upon the robustness of government initiatives, health care policies, and institutional support designed to facilitate and encourage DV screening and intervention [3].

The need for DV screening was heightened during the COVID-19 pandemic due to an identified increase in DV and gender-based violence (GBV) [4-6]. The New York State Public Health Law (NYSPHL) was amended to add Section 2805-z, which establishes hospital policies and procedures for addressing DV. The NYSPHL Section 2805-z (NYSPHL§2805-z) went into effect on December 23, 2020 [7] (refer to Multimedia Appendix 1 for complete language on legislation). Subsequently, in August 2021, the New York State Department of Health issued a “dear administrator letter” to hospital chief executive officers that included a description of NYSPHL§2805-z as well as guidance for developing a model policy. The model policy is “meant to serve as a guide for hospitals to adapt to their own format, with more specific institutional procedures,” and “provides an example policy that is in compliance with PHL§2805-z.” The model policy recommends making it a requirement for the hospital staff to administer DV screening as a method for health care providers to identify patients experiencing DV and comply with NYSPHL§2805-z [8].

However, successful adaptation and implementation of the model policy requires multidimensional support in order to achieve the goal of reaching all patients with a successful DV screening. While the model policy addresses telehealth privacy concerns and patient rights, it does not adequately address screening for DV during a telehealth visit or provide guidance on whether and how such screening should be done. In addition, although the model policy offers example questions, health care providers can use to assess a patient’s privacy and safety, it fails to acknowledge that patients must first be in a safe and private environment to accurately disclose whether they feel safe and comfortable speaking. The model policy also lacks specific directives on how to adapt the screening processes to different types of practices or specialty health care providers, both for in-person visits and telehealth care delivery.

Currently, DV screening policies in health care settings vary by state. Few states have policies requiring DV screenings and protocols, and only 19 states have laws requiring training on DV for health care providers [9]. With increasing health information exchange and patients seeking care in bordering states, variable state laws can impact patient care [10].

Health care providers play a key role in screening for DV, which is a complex process involving various factors, including people, workflows, and human-system interfaces. Therefore, it is essential to study the DV screening process not only by its individual components but also by how these components interact with each other. The sociotechnical model, which focuses on the impact and interdependencies of technical, environmental, and social factors on preventable health-related adverse events, provides a useful framework for this purpose [11].

### Aim

The aim of this study is to identify and explore challenges that health care providers face during DV screenings while complying with the requirements of policy NYSPHL§2805-z via analysis of provider interviews using an adapted sociotechnical framework. The findings informed a set of actionable recommendations to promote effective, legally compliant DV screenings in person and in telehealth health care settings.

## Methods

### Overview

This study was conducted in partnership with the New York City Mayor’s Office to End Gender-Based Violence (NYCENDGBV). The study was conducted and reported in adherence with the COREQ (Consolidated Criteria for Reporting Qualitative Research) guidelines for qualitative research. The primary focus of this study was to explore factors related to a successful DV screening in a hybrid setting of both in-person and telehealth visits through semistructured interviews with health care professionals. We describe the 2 phases of this qualitative study. In phase 1, we conducted semistructured interviews with health care professionals to explore how DV screening is implemented in both in-person and telehealth settings. In phase 2, we conducted a follow-up focus group and survey with health care stakeholders to evaluate and refine a set of recommendations derived from the interview findings. All researchers who participated in this study are trained in trauma-informed research methods. This research was conducted in compliance with the institutional review board of Cornell University.

The sociotechnical model, used as a framework for this study, is organized into *5 interconnected tiers*, including individual-level characteristics and broader system-level influences. The tiers represent critical components that collectively contribute to effective DV screening.

**Figure 1:**
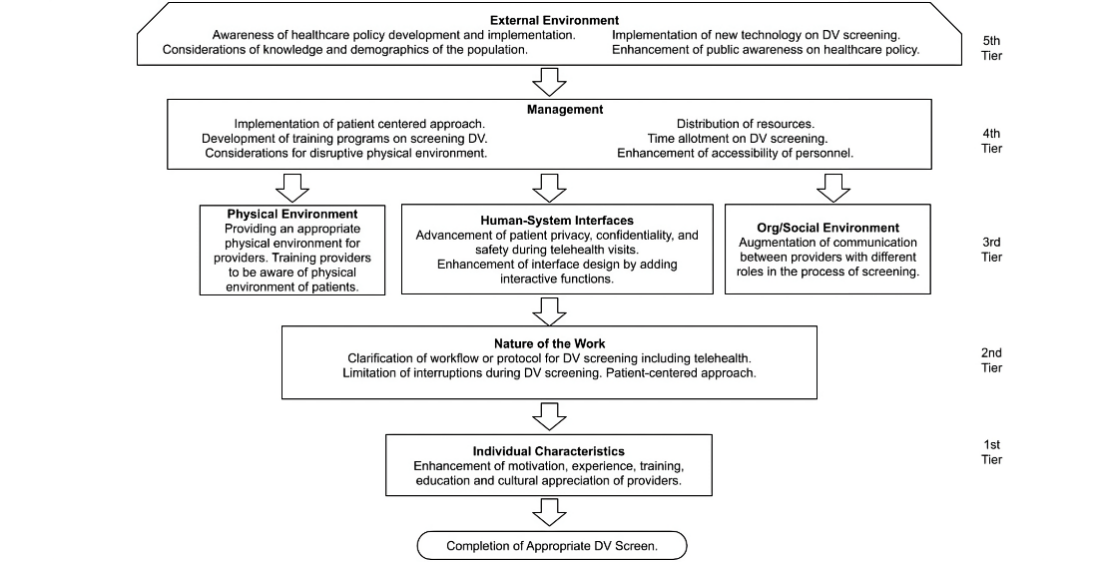
Adaptation of the sociotechnical model to identify tiered factors in successful domestic violence (DV) screening in hybrid telehealth and in-person settings.

The sociotechnical model is currently referenced by the Centers for Disease Control and Prevention to increase knowledge of organizational and personal strategies to promote a safe work environment [12]. This model has been used to inform studies on technologies used in health care, such as electronic health records (EHRs), telehealth, and mobile health tools [13]. We adapted this model to identify potential sources of support throughout institutional subsystems, with a focus on human-system interfaces and the physical environment, to inform recommendations aligned with the in-person and telehealth model, in our efforts to generate recommendations for successful DV screening (Figure 1).

### Tiers

#### Fifth Tier: External Environment

This tier reflects broad influences, such as health care policy, public awareness campaigns, and the integration of new technologies in DV screening. These external factors set the overarching context for hospital-level efforts, ensuring alignment with societal and systemic priorities.

#### Fourth Tier: Management

This tier examines the administrative considerations that influence DV screening. This includes the allocation of resources, availability of trained staff, and the development of comprehensive training programs. Management also plays a pivotal role in establishing an organizational structure that prioritizes safety, accessibility, and time-sensitive responses to screening needs.

#### Third Tier: Physical Environment, Human-System Interfaces, and Organizational and Social Environment

This tier explores how the interplay among the physical and digital environments, human-system interfaces, and organizational environments impacts patient-provider communication, care management, and patient safety to foster effective DV screening, thereby highlighting the role of human-system interfaces and workplace dynamics in fostering effective screening practices. Human-system interfaces used to communicate between provider and patient during remote clinic visits and in the documentation in the EHR, such as telehealth, change patients’ physical environment, affecting patient privacy and safety during a DV screening. Organizational culture, group norms, and communication among staff are essential for promoting collaboration and ensuring that all health care providers understand their roles in the screening process. By strengthening these technological and organizational factors, hospitals can create a more supportive environment for DV screening. Hybrid care that incorporates both telehealth and in-person care has become a common format of care. This leads to clinic sessions that include both in-person and remote care.

#### Second Tier: Nature of the Work

This tier addresses the operational aspects of DV screening. It emphasizes the need for clear workflows and protocols to guide the screening process, especially in telehealth contexts where patient privacy and safety require additional attention. This tier also recognizes the challenges posed by interruptions and competing tasks, which can undermine the consistency of screening efforts.

#### First Tier: Individual Characteristics

This tier focuses on the traits and competencies of individual health care providers. Factors such as motivation, experience, training, education, and cultural sensitivity are crucial for initiating and maintaining effective DV screening processes. These individual-level qualities form the building blocks for higher-level interventions.

### Phase 1

#### Study Design

To evaluate participants’ awareness and understanding of the NYSPHL§2805-z and its associated model policy, they were first asked whether they were familiar with the policy and its requirements. After responding, each participant was provided with a copy of the model policy to review and reflect on during the interview. This choice of study design allowed us to identify surface not only participants’ baseline policy awareness but also how they interpreted the policy’s feasibility and alignment with the lived conditions of clinical care. This reflexive, trauma-informed approach recognized that successful screening requires more than procedural compliance; it depends on whether screening can be delivered in a way that respects patient agency, anticipates power asymmetries, and adapts to environmental and technological constraints. The insights gained from this study informed the development of recommendations for conducting a successful DV screening (phase 1).

#### Semistructured Interview Guide

The semistructured interview guide for this study was developed using an iterative approach [14]. Initial questions were informed by the study’s aims and a review of relevant literature. The guide was reviewed and refined by our multidisciplinary research team with expertise in psychology, pediatric care, qualitative research, social sciences, and representation from the NYCENDGBV. This ensured content relevance and addressed participant sensitivities. Revisions were made throughout data collection, allowing for the emergence of themes and the refinement of interview questions in response to preliminary findings. Questions in the interview guide addressed issues, such as knowledge of the New York State screening policy, the nuances of screening for DV remotely, and the use of telehealth in the screening process. Example questions included (1) “What do you know about the policy?” (2) “Do you conduct visits through telehealth?” (3) “Which telehealth tools do you use to connect with patients?” and (4) “Do you take any steps to identify patients with domestic violence concerns?”

#### Participant Recruitment

This study used a purposive sampling strategy. The participants were recruited from hospitals and health organizations throughout the 5 boroughs of New York City. The NYCENDGBV circulated the recruitment flyer to health care providers across New York City. The recruitment materials targeted health care providers with experience delivering telehealth or tele–mental health services to adult patients. Social workers, who play a critical role in providing mental health support and were heavily involved in the expansion of telehealth services during the pandemic, comprised most of the participants in this study.

A total of 22 participants took part in interviews lasting approximately 60 minutes. This sample size was determined to be sufficient due to data saturation, as thematic consistency was observed across interviews, and we no longer identified new themes emerging from the interviews. Participants represented 22 different institutions and included 45% (10/22) social workers, 18% (4/22) nurse practitioners, 14% (3/22) nurses, 14% (3/22) psychiatrists, 5% (1/22) emergency room physician, and 5% (1/22) internist. In total, 32% (7/22) participants were affiliated with public organizations, and 68% (15/22) participants were affiliated with private organizations, as presented in Table 1.

**Table 1:** Characteristics of interview participants (N=22).

Participant ID	Participant role	Patient population	Organization type	Knowledge of NYSPHL§2805-z^a^ model policy	Specialized in DV^b^ care
P1	Psychiatrist	Adults and DV survivors	Public	No	Yes
P2	Physician	Adults	Public	No	No
P3	Nurse	Adults and youth	Public	Yes	Yes
P4	Nurse practitioner	Adults and youth	Private	Yes	Yes
P5	ER^c^ physician	Adults	Private	Yes	Yes
P6	Social worker	Adults, youth, and DV survivors	Private	Yes	Yes
P7	Nurse	Adults, youth, and DV survivors	Private	Yes	Yes
P8	Nurse practitioner	Adults and youth	Private	Yes	Yes
P9	Nurse practitioner	Adults and youth	Private	No	No
P10	Social worker	Adults and youth	Private	No	No
P11	Nurse practitioner	Adults and youth	Private	No	No
P12	Social worker	Adults	Public	No	No
P13	Psychiatrist	Adults	Private	No	No
P14	Social worker	Adults	Private	No	No
P15	Social worker	Adults and youth	Private	No	No
P16	Social worker	Adults	Public	No	No
P17	Social worker	Adults and youth	Private	No	No
P18	Social worker	Adults and youth	Private	No	No
P19	Social worker	Adults	Public	Yes	Yes
P20	Psychiatrist	Adults	Public	No	No
P21	Nurse	Adults and youth	Private	No	Yes
P22	Social worker	Adults and youth	Private	No	No

^a^NYSPHL§2805-z: New York State Public Health Law Section 2805-z.

^b^DV: domestic violence.

^c^ER: emergency room.

In total, 9 (41%) of the 22 participants had specialized training and experience working with DV or sexual assault survivors. These roles included a sexual assault nurse examiner, a forensic nurse examiner specializing in legal case care, an obstetrician-gynecologist social worker, and other health care providers who primarily treated patients who have experienced DV. Some of these participants were also actively engaged in DV education, training, and research. In terms of organizational roles, 5 (23%) participants held leadership positions and were instrumental in developing policies related to telehealth, DV screenings and responses, and hospital or clinic workflows.

#### Data Collection

Data collection occurred between March 2023 and July 2023. Interviews were conducted by the first author via a secure videoconferencing platform. Each interview lasted approximately 60 minutes and was audio recorded with the consent of the participants.

#### Data Analysis

We conducted a comprehensive analysis of the interviews using both deductive and inductive thematic analysis. Our deductive analysis was informed by the sociotechnical model, which provided a framework for developing a predetermined set of codes reflecting various tiers of influence within the model [15]. Concurrently, our inductive analysis allowed us to uncover additional themes and codes emerging directly from the data, ensuring a nuanced understanding of the participants’ experiences.

Our codebook comprised 63 codes. Examples of these codes include “safety,” “management—resource availability,” “human system interfaces—controls and displays,” and “individual characteristics—knowledge and skills.” Four members of the research team collaboratively developed and applied the codebook across 3 iterative rounds of coding. In round 1, all coders independently coded a shared subset of transcripts to pilot the initial codebook, compare interpretations, and refine code definitions. In round 2, the full data set was double coded using the revised codebook, with each transcript assigned to 2 researchers to ensure consistency. Discrepancies in code application were documented and resolved through weekly consensus meetings. In round 3, coders conducted a final review to apply inductive codes that emerged later in the process and to verify consistent code use across transcripts. All coding was conducted using Dedoose, a cloud-based qualitative analysis platform that supported collaborative tagging, version control, and team memoing. The research team met regularly to review the codebook and code applications and iterate on the analysis. Final themes were developed through structured synthesis and organized into 5 overarching categories aligned with the tiers of the sociotechnical framework. On the basis of our data, we developed a draft set of “recommendations for successful DV screening*.*” These recommendations were sent to participants for comments and member checking; there were no disagreements with the information included in the recommendations. We then conducted an independent evaluation of the recommendations, which we describe in the Phase 2: Study Design and Procedure section.

### Phase 2

#### Study Design and Procedure

**Figure 2:**
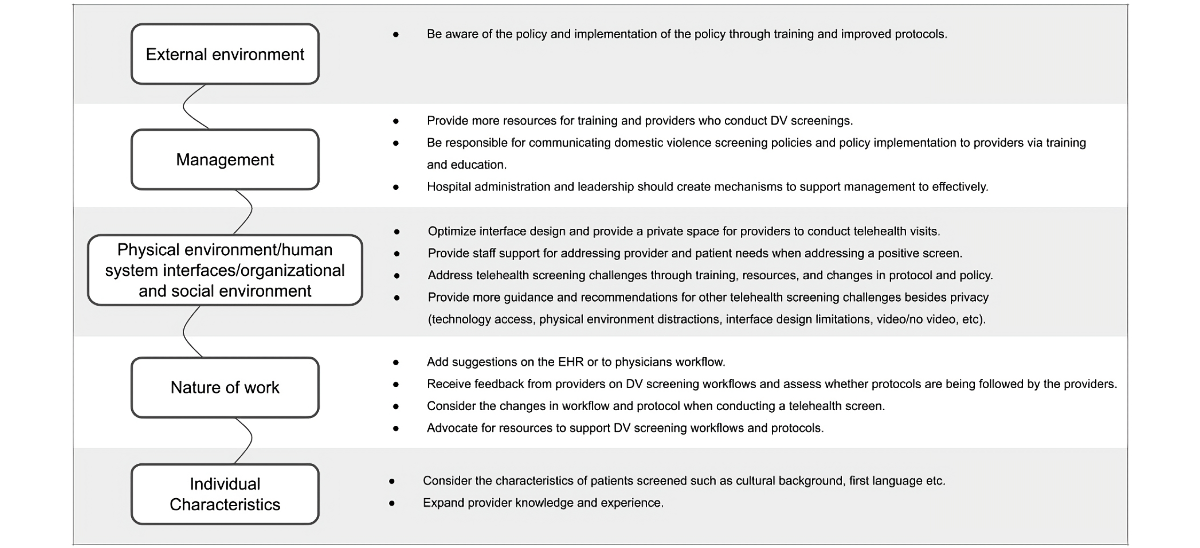
Recommendations for successful domestic violence (DV) screening. EHR: electronic health record.

After our initial data analysis and development of the “recommendations for successful DV screening” (Figure 2), focus groups and a survey study were conducted to evaluate the practicality and applicability of the recommendations derived from our findings. This second phase aimed to gather feedback from health care professionals and iterate based on it. Participants were provided with a copy of the policy and the recommendations for a successful DV screening (Figure 2) and asked to provide detailed feedback on the recommendations (Figure 2 and Textbox 1).

**Recommendations by sociotechnical tier.**

**External environment**

- There should be an increasing public awareness of domestic violence (DV) as a health issue.
- Awareness regarding and implementation of the policy through training and improved protocols are recommended.
- Hospital guidelines that align with policy implementation should be created and established.
- It is important to review the actual definition of DV screenings according to the New York State Public Health Law Section 2805-z and model policy.
- Providing guidance on resource allocation and incorporating DV screening into the health care provider workflow and the electronic health record are important.

**Management**

- Management should provide more resources for training and health care providers who conduct DV screenings.
- Management is responsible for communicating DV screening policies and policy implementation to health care providers via training and education.
- Hospital administration and leadership should create mechanisms to support management effectively.

**Physical environment, human-system interfaces, and organizational and social environments**

- There is a need to address telehealth screening challenges through training, resources, and changes in protocol and policy.
- In the model policy, more guidance and recommendations are needed for other telehealth screening challenges besides privacy (eg, technology access, physical environment distractions, interface design limitations, video or no video).

**Nature of the work**

- Hospitals should receive feedback from health care providers and survivors on DV screening workflows and when health care providers are conducting a telehealth screening to assess whether protocols are being followed by the health care providers and whether they are working well.

**Individual characteristics**

- Health care providers should familiarize themselves with the characteristics of patients screened, such as cultural background, and should stay up-to-date with supportive language choices, such as the first language, etc.
- Health care providers should become advocates for resources to support DV screening workflows and protocols.

#### Participant Recruitment

For this second phase, professionals were recruited between November 2024 and December 2024. Recruitment efforts targeted health care professionals and related stakeholders through outreach to health care organizations and professional networks in a large urban center. Participants included physicians specializing in psychiatry, epidemiology, women’s health, and neurology; social workers; psychologists; lawyers specializing in GBV; government advocacy representatives; and emergency responders, including emergency medical service and fire department personnel. Recruitment efforts targeted individuals with direct experience in DV screening through professional networks and health care organizations. Of the 29 participants, 24 (83%) took part in 1 of the 4 focus groups (6-7 participants per session lasting 75-90 minutes), and 5 (17%) participants provided feedback via a survey.

#### Data Collection and Analysis

All participants provided consent to participate in this research. Feedback was gathered from 29 professionals across various disciplines through 4 virtual focus groups and a structured survey. Participants were given the full policy document alongside the visual and textual representation of the recommendations (Figure 2 and Textbox 1). To ensure structured and meaningful engagement with the material, all participants responded to a standardized set of prompts designed to elicit specific, actionable feedback. These prompts asked participants to assess each recommendation’s clarity, feasibility, and alignment with existing workflows. They were also encouraged to identify potential barriers to implementation and suggest adaptations for their particular institutional contexts.

Participants annotated physical or digital copies of the recommendations, submitted written responses, and engaged in moderated discussions (for the focus group) to highlight surface nuanced reflections. This feedback was then thematically analyzed and used to revise and strengthen the final set of recommendations.

We discuss the phase 2 insights in the Discussion section of this paper.

### Ethical Considerations

This study was evaluated and approved by the Cornell University Institutional Review Board (IRB) of record (0144348). All participants provided electronic informed consent using an IRB-approved consent form. Interviews were audio recorded, professionally transcribed, and deidentified. Transcripts contained no identifying information and data were stored on secure, encrypted institutional servers. Participants were offered a US $50 gift card as compensation for participating in the study. This study adheres to the COREQ reporting guideline (Multimedia Appendix 2).

## Results

### Overview

We describe our findings from our interviews with 22 participants, as this was the primary focus of our research. Our findings underscored the complexity of DV screening processes and the critical need for holistic strategies to address challenges in successful screening. We found myriad factors that influenced the success of DV screenings and categorized them into themes according to the 5 sociotechnical tiers, as outlined in Figure 1: external environment; management, physical environment, human-system interfaces, and organizational and social environment; nature of the work; and individual characteristics situated within the sociotechnical framework. The themes illustrated the intricate interplay among health care policies, public awareness, resource allocation, telehealth challenges, and health care provider competencies. We found that only 7 (32%) of the 22 participants were knowledgeable about the model policy, all of whom had specialized experience working with patients who have experienced DV (Table 1).

### Theme 1: External Environment

#### Awareness of DV and the New York State Screening Policy

Variability of policy knowledge can impact successful DV screening and can be influenced by external factors (such as community awareness through advocacy efforts and public health outreach).

To investigate the awareness of recent health care policy, participants were asked if they were aware of and understood the model policy. Each participant was then provided with a copy of the policy to review. There was variability in health care provider knowledge of the model policy. In total, 16 (73%) of the 22 participants were not aware of the model policy, 1 (5%) had knowledge of the model policy but not its details or implementation, and 5 (23%) were familiar with the model policy:

I’m very familiar with, sort of legislatively and politically, everything that has happened both from the national and the statewide level but not the policy wording or implementation.

P7; nurse

However, a few participants (P10; social worker and P21; nurse) mentioned that while they did not know the specific details of the model policy, they observed the effects of policy implementation from the management level:

Well, I haven’t seen it, but I’m pretty sure that they do have that. I don’t know what it looks like exactly, or what the actual questions are, but I’m like 99% sure they are there.

P10; social worker

Another external factor that can influence the success of DV screening is public awareness of DV. A social worker described the curriculum they developed for schools and how increasing awareness of DV reporting and the services provided can encourage people to reach out for help:

I’ve developed curriculum aligned with the Enough is Enough law, focusing on Title IX, sexual violence prevention, consent, and bystander intervention—topics most requested by schools. In my workshops I tell the students, staff and/or faculty that if they’re experiencing anything like this, or know someone, they can reach out to our program directly, because we’re a confidential partner for most of these schools. I’m not so much screening in that setting, but I do have people come and ask for support afterwards.

P6; social worker

#### Policy Implementation and Guidelines

A recurring theme was the critical importance of not just having policies in place but ensuring their effective implementation within hospital settings. One participant emphasized that the real challenge lies not in the policy itself but in “how that policy actually gets implemented” (P2; physician). This underscored the need for practical strategies that translate policy into action, ensuring that health care providers can apply these guidelines consistently and effectively in their daily work.

Moreover, some participants observed notable differences in how DV screening policies are implemented across different states, highlighting the impact of state-specific policy implementation on the screening process for patients and health care providers:

I see patients in New Jersey. I don’t think they’re afforded the same confidentiality as in New York. In NJ these programs are run out of the State Attorney General’s office, and then it trickles down in each county through the county prosecutor’s office. So those programs are more focused on the prosecution than in New York where we’re based. Since we are healthcare providers we work solely under public health law and first and foremost we are concerned about the patient’s health and wellbeing.

P3; nurse

Participants noted the impact of other sections of the 2805 NYSPHL, including the protection of receiving medical care independent of law enforcement involvement:

DV patients will get an advocate in the hospital if they want one. There’s other public health laws that are part of that whole 2805 series. But you’ll look at 2805-i and 2805-p more specific to sexual assault. Patients can unconditionally get medical care, whether they involve law enforcement or not, they may not be prepared to involve law enforcement. As for healthcare providers, we will provide healthcare unconditionally.

P11; nurse practitioner

### Theme 2: Management

#### Resource Allocation for Health Care Providers

The allocation of resources, including staffing levels, translation services, and the management of patient load and scheduling, plays a critical role in the effectiveness of DV screenings. This resource allocation directly impacts health care providers’ ability to conduct thorough screenings and provide comprehensive follow-up care. This was noted by many of the participants. For example, a participant emphasized the importance of adequate staffing, noting that the availability of a well-supported team enables clinicians to provide comprehensive care:

Ultimately, the therapist that a patient is assigned to can rely on additional support from their supervisor if needed. When it comes to navigating legal issues or accessing additional services, case managers are available to assist, ensuring that clinicians are never left without the necessary support to care for their patients.

P14; social worker

#### Challenges Due to Patient Volume and Limited Time

However, the reality of clinical practice often contradicts these ideals, creating significant barriers to effective screening. Participants expressed significant concerns about the impact of high patient loads and time constraints on their ability to provide effective screening and comprehensive care. These structural limitations created a fundamental tension between the goal of thorough DV assessment and the operational pressures of maintaining clinical schedules. One participant, a social worker, highlighted the frustration with time constraints, noting that the desire to extend sessions for more comprehensive care often conflicts with the need to maintain schedules. Time constraints and high patient volumes can restrict the ability to address each patient’s unique circumstances. They explained as follows:

If I extend the time, I may have to cancel other appointments, or everyone else would be late, which isn’t fair to the other patients. Unfortunately, we don’t always have the time that I think is necessary.

P16; social worker

Timing posed a particular challenge when conversations about DV surfaced near the end of a session. Health care workers described that this could make it difficult to adequately address the situation and develop a safety plan in the time available. This timing challenge represented a critical mismatch between clinical workflow design and the unpredictable nature of sensitive disclosures. A social worker told us the following:

Our schedules are incredibly tight, with sessions booked back-to-back. Unfortunately, DV-related questions often surface toward the end of a session, and developing a proper safety plan can take 20 to 30 minutes. If I’m already heading into my next session, it’s impossible to dedicate the necessary time. In those cases, the safety plan might need to be delayed until the next day or later in the week. It’s challenging for both the patient and me to carve out the time and secure a safe space to work through these critical details.

P18; social worker

#### Guidance and Training for Health Care Providers

Beyond staffing and time constraints, participants identified significant gaps in structured support and systematic guidance for DV screening implementation. Multiple participants noted that more guidance, structured support, and training were needed for successful screening processes:

I feel like maybe it could be beneficial to have maybe more guidance or a structured approach to doing so.

P15; social worker

#### Competing Clinical Responsibilities

The challenge of DV screening becomes more complex when considered within the broader landscape of competing clinical responsibilities. Participants noted additional demands on their time that impacted their ability to complete a thorough screening. This multiplicity of required screenings creates what can be characterized as “screening fatigue” among health care providers, where the sheer volume of mandated assessments becomes overwhelming:

So I think education and guidance certainly is part of it and there’s structural factors that make it very challenging to fit in all the assessments that should be done in a 20-minute visit in which the patient has shown up late or you’re double-booked, and then there’s 10 people waiting in the waiting room. There’s domestic violence screening, depression and anxiety screening and social determinants of health screening. There’s smoking cessation discussions, alcohol use, recreational drug use, safe sex, preventive…?

P2; physician

These findings revealed that structural barriers fundamentally undermined the effective implementation of DV screenings. Participants highlighted how limited time, high patient volumes, and competing demands impacted effective screening and safety planning. These challenges underscored the need for comprehensive structural reforms rather than provider-focused solutions alone, including better scheduling flexibility, enhanced organizational guidance, and systematic support systems to enable meaningful and timely care. Without addressing these systemic barriers, even well-intentioned policies and well-trained health care providers will struggle to achieve optimal screening outcomes.

### Theme 3: Physical Environment, Human-System Interfaces, and Organizational and Social Environment

#### Unique Challenges of Telehealth

The use of human-system interfaces during telehealth DV screenings requires careful consideration of the physical environment, technology resources, and strategies to protect patient privacy for a successful screening. However, participants found that these considerations can be challenging to address.

A primary concern expressed by participants was safeguarding patient privacy during telehealth sessions. In face-to-face interactions, health care providers can directly ensure confidentiality by asking others to leave the room. However, telehealth complicates this dynamic, as health care providers cannot verify if a patient truly has privacy. This uncertainty can undermine questions about and disclosure of sensitive information essential for effective DV screening:

In person, I can just say, “Hey, can you leave the room? Do you mind letting me continue this interview without you?” But in telehealth, we don’t always know if the patient even has privacy. They tell us, “Yeah, I have privacy,” but I don’t know if maybe the person is listening to them in the other room or recording.

P16; social worker

Participants also pointed out that patients do their best under the various constraints that they experience. Many patients live in shared spaces or have schedules that make uninterrupted time difficult to secure. One participant noted the following:

Patients can’t always find somewhere quiet to talk. We want it to be as safe as possible for the patient, but we have to recognize that some of our patients are under various constraints. If six people are sharing a one bedroom apartment, that person’s working with what they got.

P19; social worker

In addition to confidentiality, safety was also an important consideration when conducting a DV screening using telehealth. Many participants emphasized the challenges of ensuring a patient’s safety remotely:

Often, the first step…is setting the scene and…asking the patient if they’re in a quiet space, if they feel comfortable and are safe to talk. But that’s not necessarily a widely adopted practice, based on what I’ve seen. How do you know when they’re not safe? If they are truly in an unsafe environment and somebody is on the other side of a screen or out of camera view and they hear the question, what else are they going to say aside from yes?

P2; physician

Looking at the technical aspects, interface design can also impact the success of telehealth screening. A social worker emphasized the importance of interactive functions and present limitations:

I think some interactive functions are very important, just sharing the screen. That can be helpful when I’m doing psychoeducation, playing a video with them or just having them read through the documents. But for Epic [Epic is an EHR], so that is a different story. “We can do this with you, but we can’t share the screen.” They have a function, but it never works for me.

P18; social worker

The absence of a chat functionality on telehealth platforms was highlighted as a significant challenge during DV screenings:

Well, I think at a basic level, good audio and video quality is important. I’m not a techie, we don’t have the chat feature within our video platform that we use for our visits. If we had it and I wanted to share a resource, at that moment, I could send it to them. especially for a DV situation.

P22; social worker

Distractions in the surrounding environment of the patients also arose as a screening challenge that can affect the success of telehealth screening:

Oftentimes, there’s distractions during those televisits, clients that maybe they’re trying to cook for their family or clean. Or I’ve had clients order takeout, and they had to run downstairs to pick up the food delivery. And so it kind of takes away the focus from the session.

P12; social worker

However, another social worker highlighted that patients come from different circumstances and thus it is difficult to expect patients to be in an ideal situation while participating in a telehealth screening:

Telehealth mental health services is a real challenge because patients can’t always be somewhere that’s quiet, or private. You might catch someone for a therapy session when they walk to work or they are in the break room or bathroom and they’re carving out time where they can. So, ideally for us, we want it to be as safe and sound but we also have to recognize that our patients are doing the best they can.

P19; social worker

Challenges related to patients’ difficulties with technology access were frequently mentioned by participants. These challenges included issues with internet connection, a lack of patient knowledge about using telehealth, and sometimes limited access to technology:

There’s definitely some communication issues going on there. Sometimes with technical difficulties where someone has a hard time turning on their speaker, or the service is so bad, it’s freezing. Either I’m getting frozen half the time I’m speaking, or the patient is. That’s frustrating too.

P9; nurse practitioner

Another health care provider noted the impact of these issues, explaining how troubleshooting technical problems can consume time available for the actual visit:

From the clinician’s perspective, if you log into the electronic health record and you go into the video room and nobody’s there, you have to troubleshoot. You have to call the patient and say, “Hey, I’m in the video room. How can I help you get in?” And then you spend five minutes on tech support, and then the audio isn’t working on the video, or the video isn’t working, or things are getting choppy. You’ve started the visit 10 minutes behind of your 20-minute visit, and you’re also running 40 minutes behind in your overall schedule. That’s much more of a headache than a phone call.

P2; physician

These experiences underscored the significant impact of technical barriers on the quality and timeliness of care, highlighting the need for more reliable and user-friendly telehealth solutions that can better support DV screenings and other critical services.

Adding to the technology access problem, a participant emphasized the particular challenges faced by older patients in engaging with video platforms and advocated for the need to address these issues:

I think one of the things that we all wish for is just ease of use for patients. We find that our older patients have a hard time with technology. They’d rather just call us. The video feels very overwhelming to them. I wish that was a little bit easier for them to navigate.

P19; social worker

The importance of asking patients to turn on their video during the telehealth visit was often highlighted because health care providers had a hard time understanding their situations and providing good quality treatment:

I usually also will provide some psychoeducation around why it’s important to have the cameras on to be able to see body language and facial expressions. Those things are really crucial for the therapy process. I personally really hate doing phone sessions because I feel like it’s so much harder, because we can’t see the person, but we do try to be flexible. I know that some other folks have clients and meet them by phone, because of safety concerns or it’s easier for them if they’re in a domestic violence shelter.

P6; social worker

A participant shared the experience of requiring patients to turn on their camera, with only limited exceptions, because of its importance in understanding their situations:

So I demand they turn on their camera. I set that expectation when I do the outreach, I’ll be like, “Hey, we’ll be on video. Because if I can’t see you, I don’t really know what’s going on.” But there were a few times for my ongoing patients that they didn’t feel comfortable turning video on, maybe because they think they look like a disaster. There were a few times I allowed them to just be audio, but on very rare occasions and definitely not first-time patients.

P17; social worker

#### Features of the EHR

EHR systems can play a critical role in supporting effective DV screening by offering built-in features that streamline health care provider awareness and triage and can impact a successful screening. A specific tool that allows health care providers to color-code patients by their assessment helps them provide care. A participant stated the following:

Because we were able to make this tool in Epic, we were able to flag that category as basically green, yellow, or red, and that state, in that assessment, in that chart of, “Everything’s fine,” or “Maybe there’s some concerns,” or red. So if the provider reads a note they see in red, there’s a section that you better watch out for…It might be orange, because this partner isn’t abusive, or because this partner isn’t violent now, but there is some history so we want to keep an eye out.

P19; social worker

### Theme 4: Nature of the Work

#### Health Care Provider Strategies for Effective Screening

The nature of clinicians’ workflow, including standard screening processes and role delineation, plays a critical role in the successful implementation of DV screenings. Compliance with government policies is essential, yet the workflow’s structure can significantly impact the outcome of these screenings.

Participants described a variety of approaches to DV screening, indicating variability in how these processes are implemented across different health care settings. One participant noted that during initial assessments in the emergency room, there are required questions about intimate partner violence, safety in the home, and other safety concerns:

I feel like I just know that in the initial assessment, when people come into the emergency room or the hospital, I know that there’s questions that they’re required to ask. And I believe that those include questions on intimate partner violence, safety in the home, sexual violence, suicidal ideation, all of those safety concerns.

P6; social worker

Even when there was a screening protocol that was generally followed by the health care providers, frequently, the screening did not meet the practices mandated by the policy. For example, the policy might mandate something, but the provider would actually engage in a less rigorous process:

We’re so busy sometimes that we are three patients behind. We don’t have time to go through and make sure that every single questionnaire has been answered. Sometimes it’s really easy just to copy and paste what you wrote three months before and ask if there’s any changes to their history, without specifically asking about if they’re in a new relationship, or if their relationship now feels unsafe, than before. Also for the people who don’t feel comfortable talking about it too, I think that they may very much lie to me and I wouldn’t even know.

P9; nurse practitioner

Another participant described a more nuanced approach to screening, especially in pregnancy assessments where sensitive topics are introduced gradually, leading to higher rates of disclosure:

So in the pregnancy assessments that I would do, it’s built into a very careful conversation. You ask a bunch of other questions first that are less charged. And you slowly warm up to, “And how is the person that you became pregnant with? How do you guys get along in general? Can you tell me a little bit about what that looks like when there’s conflict between the two of you?” Then we always say, “At any point in the relationship have things ever gotten physical between the two of you with hitting, holding, or shoving, you felt unsafe in any other way?” And we ask about controlling behaviors. I think that’s actually why we had such a high rate of disclosures. I would hear all the time, “Well no, it’s not domestic violence. He doesn’t hit me.” “He just said if I don’t do this, this, and this, he won’t sponsor me.” It was like, “Oh, wait a minute.”

P19; social worker

P19 described a strategic and relational approach to DV screening, particularly in the context of pregnancy assessments. The health care provider begins with “less charged” topics, building conversational trust before transitioning into more sensitive areas. This scaffolded questioning strategy reflects trauma-informed care principles emphasizing emotional safety, trust building, and patient empowerment. By the time the health care provider introduces direct questions about physical violence or control, the patient is more prepared to reflect critically on their experiences. Critically, the example revealed how patients often minimize or misclassify abuse based on a narrow definition of DV as physical harm (“He doesn’t hit me”). The social worker’s recounting of a patient who dismissed coercive immigration threats as “not domestic violence” highlighted a key challenge in DV screening: patients may not recognize nonphysical control, such as threats to immigration status, as abuse, thus calling for more public awareness regarding DV.

In addition, this underscored the need for screening protocols that explicitly address coercive control and structural vulnerabilities (eg, immigration dependency), especially during pregnancy, a time when patients may have heightened contact with the health care system but also increased vulnerability.

#### Positive Screening Responses

Another important part of the DV screening workflow was the positive screening response that described what health care professionals did when a positive screening occurred, affirming the patient was in a DV situation and the importance of providing support, resources, and information to patients:

The minute somebody discloses that they’re in a DV relationship, it’s our job to get them into a shelter and out immediately…Safety planning is a very daunting aspect for a lot of folks. The biggest challenge for staff is making sure that we give that person all the resources we can, and being mindful that they’re going to make their choice. It’s our role to be there for them regardless of their choice, safely flag that chart, make sure that we can try to sneakily check in with them next time they come, or that they have everything they need so if that day comes and they need to make a different decision?

P19; social worker

The screening process workflow for telehealth requires an even more nuanced approach because of the limited situational awareness noted earlier and the challenges of building trust and ensuring patient safety in telehealth exchanges. Some participants stated that there is a need for creating protocols specifically for telehealth screenings:

Yeah, I think for telehealth, people get very anxious because they don’t know how to do it, in what order, what to do if somebody says yes. I paired up with a team of social workers, and tried to come up with a trauma-informed approach. We tried it on our colleagues, where to put the question, we did trial and error. And a lot of people gave us feedback saying that patients say, “Oh, I’m just calling for my finger and they’re asking me upfront about domestic violence. It feels funny.”

P5; emergency room physician

### Theme 5: Individual Characteristics (Health Care Provider Familiarization With Patient Characteristics)

Clinicians’ understanding of patient considerations, such as trust and engagement, language concordance, cultural considerations, and demographic considerations, can impact the likelihood of a successful screening.

Several participants shared that sometimes patients have a hard time trusting interpreters because they think they are not interpreting correctly, or there are things they do not want to share. Therefore, they prefer health care providers who speak their language. One participant shared the following:

It’s hard to create a space for them to feel comfortable sharing and disclosing information...I see a lot of Spanish-speaking patients. When I do work with them, either intake or therapy will say, “I’m so glad you speak Spanish because I hate using an interpreter. They don’t ever interpret correctly or there’s things that I don’t want to share.”... Individual characteristics including motivation, experience, training, education and cultural appreciation can impact the likelihood of a successful screen.

P22; social worker

A participant also mentioned that there are circumstances where patients feel more comfortable disclosing immigration status to someone who speaks their preferred language:

A lot of patients will ask for help with applying for disability because they work in the fields and they don’t necessarily want you to know that they’re undocumented. They feel more comfortable if a case worker speaks Spanish. and might tell them they’re victims because they feel that they can relate better. They’re dependent on that person, and so they don’t want to disclose or risk losing their job.

P13; psychiatrist

In addition, the knowledge and comfort level of the individual provider may also impact the success of the screening. Some participants stated that while they are comfortable asking the questions, they are not certain about providing the right resources and guidance:

I think I feel very comfortable asking the basic questions, because I’ve been doing them for so long. I think sometimes if they say yes and there’s no social worker on site, I would feel a little bit more concerned about making sure that I’m giving them all the right resources and making sure that they know where to go if they feel unsafe.

P9; nurse practitioner

## Discussion

### Principal Findings

This study explores the interrelated factors impacting the success of mandatory DV screenings in health care settings. We applied the sociotechnical model to examine the multiple and interrelated levels of complex systems that influence or inhibit the ability of a health care provider to engage in a successful DV screening [16]. Our findings demonstrate how health care policies, public awareness, resource allocation, telehealth challenges, and provider competencies are integral forces in the screening process. The complexity and interdependency of these factors underscore the need for comprehensive, integrated policy implementation, adequate resource allotment, and staff development strategies in order to improve the likelihood of a successful DV screening in a telehealth and in-person hybrid environment, ultimately providing better support for patient survivors.

By situating this work within the sociotechnical model, our research broadens its application beyond traditional uses to address DV screening. This model’s extension emphasizes the necessity of considering technological, procedural, environmental, and social factors together. By integrating these diverse elements, we achieve a more complete understanding of the complexities involved in DV screening. This holistic approach not only helps identify and mitigate potential risks but also informs the development of robust and effective strategies to enhance support for DV survivors across different health care settings.

Many health care providers in our sample were unaware of the model policy and the NYSPHL§2805-z directive and lacked knowledge of standardized procedures for implementing effective DV screenings, whether in traditional face-to-face emergency department visits or telehealth visits [7]. This suggests a communication breakdown between hospital administration, which received the NYSPHL§2805-z mandate and the model policy, and the patient-facing staff. Training staff to identify and support patients affected by domestic and gender-based violence—while prioritizing privacy and safety in digital spaces—can significantly improve the effectiveness of screening efforts.

Our findings also suggest that specific challenges in implementing the new law and the model policy in telehealth settings include (1) the lack of training and guidance for health care providers on practices related to DV and GBV; (2) difficulty navigating the telehealth, digital infrastructure, and other electronic systems, especially for at-risk populations; and (3) difficulty assessing patient situations for safety and privacy concerns in telehealth environments. There is a need for clearer policy, support, and guidance for health care providers to deliver telehealth services to DV and GBV survivors in a way that prioritizes safety, confidentiality, and privacy during telehealth visits. Critically, our findings suggest that the model policy and its subsequent implementations lack guidance on how to accommodate key sociotechnical constraints faced by patients, such as limited broadband internet access or lack of a private space for telehealth visits.

Drawing from the findings, we developed the *recommendations* *for a successful DV screening* (Figure 2) to address these challenges and provide actionable guidance for improving screening outcomes. These recommendations focus on bridging the gap between policy mandates and the realities of clinical practice, particularly within integrative care models that incorporate both telehealth and in-person visits. Some recommendations were informed directly by health care providers’ suggestions and experiences, such as enhancements to clinicians’ workflows or using supportive language and a scaffolded approach to DV screenings, while others emerged in response to challenges they encountered. Mapping these recommendations across all tiers of the sociotechnical framework (Textbox 1) can support the adoption of a holistic approach to improving DV screenings instead of confining solutions to a single level. This comprehensive perspective aligns with health care providers’ calls for structural changes that go beyond provider-level interventions. It addresses both systemic challenges, such as policy awareness and resource allocation, as well as operational barriers, including the need to ensure patient privacy and safety in telehealth environments.

While these recommendations were derived from our findings in the first phase of the study, the second phase, described in the Methods section, involved the evaluation of their practicality and applicability. The feedback was gathered from 29 professionals across various disciplines through 4 virtual focus groups and a structured survey, with all participants assessing them as valuable and useful in practice. Furthermore, their feedback addressed not only the substance of the recommendations but also the need and strategies for effectively tailoring and delivering them to diverse audiences.

Most of the focus group and survey participants’ feedback focused on training and education of health care professionals, both in assessing DV and responding to disclosures. Survey respondents and focus group participants alike highlighted the need for more specific, actionable training on handling disclosures, including simple, empathetic phrases, such as “Thank you for sharing that with me,” to build trust and provide emotional support.

Feedback also underscored the importance of tailoring recommendations to the intended audience. Participants suggested that administrators and frontline health care providers have distinct needs that should be addressed separately in educational training. For example, participants shared that, “administrators may require strategic overviews for implementing policy and allocating resources,” while “health care providers need step-by-step protocols integrated into their workflows.” Role-play–based training emerged as a preferred method to help health care providers practice these sensitive interactions, thereby reducing anxiety and building confidence. In addition, participants strongly recommended embedding the recommendations into training sessions to ensure they are contextualized and practically applicable.

Furthermore, feedback revealed systemic barriers, such as time and staffing constraints, which contribute to rushed screenings and hinder health care providers’ ability to establish rapport with patients. Telehealth-related challenges, including limited access to private spaces and technology among patients, were also frequently mentioned. Participants suggested future work could focus on strategies to address these specific barriers, such as providing alternative screening workflows for patients in suboptimal telehealth environments. We acknowledge the importance of this feedback and the need for future research to focus on developing educational training with government and advocacy organizations.

### Comparison With Prior Work

There are few studies discussing the effects of the hybrid model of telehealth and in-person visits and the use of telehealth on DV screenings [17,18]. Several of these have also focused on investing in health care provider training for screenings [19-21]. Very few studies have focused on state-level DV screening policies and their impact on hospital policy and practice. While these other works are allied, our paper is the first to discuss the implementation and response to NYSPHL§2805-z in New York hospitals, which are mandated to implement policies and procedures to identify patients experiencing DV.

### Limitations

There are 2 conditions that limit the applicability of our findings. First, NYSPHL§2805-z is a state-wide mandate. Our sample is primarily based in New York City, an urban center, resulting in potential limitations to the findings. It is possible that there is even less NYSPHL§2805-z and related policy knowledge in lower resource counties across New York State. This may limit the generalizability of the findings. Second, many of the participants were hospital social workers and not physicians or nurses, who may have differing perspectives. It is possible that other hospital-based personnel who are also responsible for the screening process have alternative perspectives.

### Conclusions

Our findings suggest a noteworthy association between participants’ specialized experience in DV and their awareness of the NYSPHL§2805-z and its model policy. Specifically, those with more direct experience in DV cases exhibited a higher level of familiarity with the NYSPHL§2805-z and the model policy, indicating that specialized knowledge in DV may facilitate better understanding and implementation of related health care mandates. However, this also highlights a critical gap; many health care providers without such specialized experience demonstrated a lack of awareness of the NYSPHL§2805-z and related policies, underscoring significant shortcomings in the dissemination and communication of this crucial guideline within health care settings.

In assessing the implementation and effectiveness of the model policy, we examined whether participants adhered to established protocols for DV screening and consistently applied these screenings to every patient. Despite the introduction of the NYSPHL§2805-z and the model policy, our findings reveal that there are multiple barriers to successful DV screenings that are not addressed in current DV policies. This suggests that the model policy, in its current form, is insufficient to ensure comprehensive and consistent DV screening practices.

To address these shortcomings, we developed guidelines to enhance policy awareness and improve implementation strategies, which were evaluated for practicality and applicability with a focus group and surveys. Hospital administration and leadership must take proactive steps to strengthen the communication of NYSPHL§2805-z and related policies and procedures to all health care providers, ensuring that their importance is clearly understood and integrated into regular practice. In addition, there is a pressing need for targeted education and training programs focused on DV. These programs should be designed to equip health care providers with both the knowledge and practical skills necessary to conduct effective DV screenings, particularly in complex or sensitive situations. From a sociotechnical standpoint, an intervention focused on enhancing the individual competencies of health care providers could yield substantial improvements in screening practices. Equipping staff to recognize signs of domestic and gender-based violence, while safeguarding privacy and digital safety, can make screening events far more effective.

Moreover, the integration of supportive mechanisms within health care providers’ workflows, such as prompts and reminders embedded in EHR, and the provision of support staff with specialized DV training could play a pivotal role in reinforcing adherence to policies designed to comply with NYSPHL§2805-z. Aligning hospital guidelines more closely with the NYSPHL§2805-z requirements will also be crucial, along with the establishment of feedback systems that allow health care providers to share insights and challenges encountered during DV screenings.

Furthermore, the model policy requires revisions to account for the unique challenges posed by telehealth. As telehealth becomes an increasingly integral part of health care delivery, it is vital to consider factors such as technological accessibility, the influence of physical environments, and the limitations of video interfaces. Updating policies to address these considerations will help ensure that DV screenings conducted via telehealth are as effective and reliable as those conducted in traditional clinical settings.

By addressing these identified gaps and enhancing the implementation of the NYSPHL§2805-z, health care institutions can significantly improve the quality and consistency of DV screenings, ultimately leading to better outcomes for patients impacted by DV.

## Appendix

Multimedia Appendix 1 New York State Public Health Law Section 2805-z.

Multimedia Appendix 2 COREQ (Consolidated Criteria for Reporting Qualitative Research) checklist.

## Abbreviations

COREQ: Consolidated Criteria for Reporting Qualitative Research
DV: domestic violence
EHR: electronic health record
GBV: gender-based violence
IRB: Institutional Review Board
NYCENDGBV: New York City Mayor’s Office to End Gender-Based Violence
NYSPHL: New York State Public Health Law
NYSPHL§2805-z: New York State Public Health Law Section 2805-z

## Data Availability

The datasets generated and analyzed during this study are not publicly available due to the risk to participants’ anonymity and privacy if all collected qualitative data is shared.
