# Supplementary material for: Sociotechnical Challenges in Implementing Domestic Violence Screening via Telehealth and In-Person Care: Qualitative Study on Clinicians’ Perspectives
Source: J Med Internet Res. 2025 Oct 23;27:e75244. doi: 10.2196/75244 (PMC12592885; doi:10.2196/75244)
Supplement: Multimedia Appendix 1 [file jmir_v27i1e75244_app1.docx]

## Appendix A

### Public Health Law 2805-z

Hospital domestic violence policies and procedures

Public Health (PBH) CHAPTER 45, ARTICLE 28

The New York State Public Health Law was amended to include a new Section §2805-z, regarding Hospital Domestic Violence Policies and Procedures, effective December 23, 2020. With the proper training and tools, hospital staff can play a significant role in recognizing and responding to domestic violence, and in turn, improve patients' physical and emotional health and safety, and reduce domestic violence incidents.

Pursuant to this new law,

1. Every general hospital shall:

1. develop, maintain, and disseminate written policies and procedures for the identification, assessment, treatment and referral of confirmed or suspected cases of domestic violence;
2. establish and implement a training program for all nursing, medical, social work and other clinical personnel, and security personnel working in hospital service units on these policies and procedures; designate a staff member to contact the domestic violence or victim assistance organization providing victim assistance to the geographic area served by the hospital to establish the coordination of services provided to domestic violence victims.

2. Upon admittance or commencement of treatment of a confirmed or suspected domestic violence victim, advise the victim of the availability of the services of a domestic violence or victim assistance organization, and contact the appropriate organization and request that a victim assistance advocate be provided if the domestic violence victim requests one.

3. The commissioner shall promulgate such rules and regulations as may be necessary and proper to carry out effectively the provisions of this section. Prior to promulgating such rules and regulations, the commissioner shall consult with the office for the prevention of domestic violence and other such persons as the commissioner deems necessary to develop a model policy for hospitals to utilize in complying with this section and to identify the domestic violence or victim assistance organizations operating in each hospital's geographic area, a list of which the commissioner shall provide to hospitals with the model policy.
